# Azelastine Nasal Spray in Non-Hospitalized Subjects with Mild COVID-19 Infection: A Randomized Placebo-Controlled, Parallel-Group, Multicentric, Phase II Clinical Trial

**DOI:** 10.3390/v16121914

**Published:** 2024-12-13

**Authors:** Peter Meiser, Michael Flegel, Frank Holzer, Dorothea Groß, Charlotte Steinmetz, Barbara Scherer, Rajesh Jain, CARVIN-II Study Group

**Affiliations:** 1 Ursapharm Arzneimittel GmbH, Industriestraße 35, 66129 Saarbrücken, Germany; 2 Pharmalex India Pvt. Ltd., Noida 201301, India

**Keywords:** COVID-19, azelastine, nasal spray, SARS-CoV-2, viral load, COVID-19 related hospitalization, clinical trial

## Abstract

Nasal spray treatments that inhibit the Severe Acute Respiratory Syndrome Coronavirus 2 (SARS-CoV-2) entry into nose and nasopharynx at early stages can be an appropriate approach to stop or delay the progression of the disease. We performed a prospective, randomized, double-blind, placebo-controlled, parallel-group, multicentric, phase II clinical trial comparing the rate of hospitalization due to COVID-19 infection between azelastine 0.1% nasal spray and placebo nasal spray treatment groups. The study furthermore assessed the reduction in virus load in SARS-CoV-2-infected subjects estimated via quantitative reverse transcriptase polymerase chain reaction (RT-PCR) using nasopharyngeal swabs in both groups during the treatment period. A total of 294 subjects with mild COVID-19 infection were screened and randomized in a 1:1 ratio. There was no incidence of COVID-19-related hospitalization in either treatment group. Mean virus load was significantly reduced in both groups during the 11 treatment days as compared with baseline viral load values. The reduction in virus load in the azelastine 0.1% nasal spray group was significantly higher than the reduction in the placebo group at day 11 (log_10_ 5.93 vs. log_10_ 5.85 copies/mL, respectively, *p* = 0.0041). A total of 39 (32.0%) subjects in the azelastine 0.1% treatment group and 40 (31.0%) subjects in the placebo group reported 48 and 51 adverse events, respectively. It is therefore concluded that azelastine 0.1% nasal spray is an efficacious, safe, and well-tolerated treatment of mild COVID-19 infection.

## 1. Introduction

In 2019, a new coronavirus known as SARS-CoV-2 (severe acute respiratory syndrome coronavirus 2) erupted throughout the world causing the highly contagious illness coronavirus disease 2019 (COVID-19) [1]. Multiple variants of SARS-CoV-2 have been identified, of which a few are considered variants of concern due to their propensity to increase transmissibility or virulence [2]. As per the World Health Organization (WHO), there was an increase in the number of new COVID-19 cases by 52% during the 28-day period from 20 November 2023 to 17 December 2023 as compared to the previous 28-day period, with over 850,000 new cases reported globally [3]. As of February 2024, India has witnessed >44 million cases and >0.53 million deaths due to COVID-19 [4]. India is the most impacted country, ranking second to the United States of America (USA) in terms of the number of cases, and third to the USA and Brazil in terms of deaths [5].

SARS-CoV-2 enters the respiratory tract via the nose and causes an infection through a series of events including binding to the nasal epithelial cells, entering the nasal epithelial cells via the host receptor angiotensin-converting enzyme-2 (ACE-2), undergoing local replication and transmission after entry, as well as causing the infection of ciliated cells in the upper respiratory tract [6]. Early signs and symptoms of COVID-19 infection include fever, headache, sore throat, and loss of smell and taste. In adults, a significant reduction in CD8+ and CD4+ T-cells in the primary stages of the disease when encountered with the virus is observed. The decrease in the T-cells leads to the acute respiratory distress syndrome for approximately 7–10 days which further causes the fast viral replication phase of the disease, resulting in the release of a storm of pro-inflammatory cytokines, initiating chemokine responses, and infiltration of inflammatory cells [1]. So as to prevent the progression of the disease at early infection phase wherein the viral load is highest in the nose and nasopharynx, the applicability of nasal spray with an active substance that inhibits virus entry and replication may stop or delay the progression of the disease to the lower respiratory system and reduce the transmission to uninfected individuals [7].

Azelastine hydrochloride nasal spray (concentration of 0.1% *w*/*v*) is currently an approved medicinal product to treat allergic rhinitis. Several studies have explored azelastine’s potential as a promising drug-repurposing candidate to reduce SARS-CoV-2 viral load and infection rates [8,9,10,11,12,13]. The mechanism of azelastine in exerting the antiviral effect may involve its direct action on viruses, host cell pro-viral factors, and on the virus-induced host response [14]. Azelastine nasal spray might be used as an instant approach in the initial stage of COVID-19 infection to prevent nasal colonization with SARS-CoV-2; therefore, it may have a great effect in preventing the viral spread from the nasopharynx to the lungs [13].

The first clinical dose-finding proof-of-concept study in subjects tested positive for SARS-CoV-2 was the German CARVIN trial [15]. The study results provided the first human data showing that azelastine hydrochloride 0.1% nasal spray may indeed be effective in accelerating the reduction of virus load in the nasal cavity and improving COVID-19 symptoms in subjects. Therefore, to further understand the impact of azelastine hydrochloride in treating SARS-CoV-2-infected subjects, the present phase II study (CARVIN-II) was planned based upon the positive outcome of the phase II dose-finding study [15]. The aim of this study was to evaluate the clinical safety and efficacy of azelastine nasal spray in Indian subjects who tested positive for SARS-CoV-2 with mild symptoms and not requiring hospitalization.

## 2. Materials and Methods

### 2.1. Study Design

In this multi-center, phase II, randomized, prospective, double-blind, parallel-group, placebo-controlled efficacy and safety study, non-hospitalized Asian subjects with mild COVID-19 infection, who were detected positive with rapid antigen tests (RATs), were randomized to receive azelastine 0.1% nasal spray or placebo nasal spray.

Subjects self-administered two puffs of the nasal spray, with one puff per nostril, five times daily, preferably at 3 h intervals during the day, from day 2 to day 10. Daily administration on days 2–10 was not less than 3 application per nostril. On day 1 (day of inclusion of subject), at least two puffs per nostril were administered. On day 11 (last day of the study treatment), at least one application was completed before the Investigator’s visit. All subjects were provided with standard supportive treatment as per the Ministry of Health and Family Welfare (MoH FW), Government of India [16].

The study was initiated after protocol approval from the Institutional Ethics Committee or Institutional Review Board. A full list of Ethics Committees involved is provided in the Supplementary Table S1. The study was carried out in compliance with the Good Clinical Practice, principles of the Declaration of Helsinki, and all applicable local and national regulatory requirements. Prior to the start of the study, the clinical trial was registered in the Clinical Trials Registry-India (clinical trial ID: CTRI/2022/09/).

### 2.2. Participants

Male and female subjects ≥ 18 years of age, willing to provide nasopharyngeal swabs, with a positive RAT for SARS-CoV-2 were enrolled from the general population and treated on the same day based on randomization. Subjects with blood oxygen levels nearing moderate Saturation of Peripheral Oxygen (SpO2) (<93%) were excluded.

Women of reproductive age agreeing to employ reliable contraceptive methods (including hormonal contraception, barrier methods, or abstinence) until day 11 or those unable to bear children were enrolled. Pregnant or lactating women were not included in the study. All inclusion and exclusion criteria are listed in (Supplementary Table S2). Subjects were required to possess the ability to give informed consent and self-administer the nasal spray.

### 2.3. Randomization and Masking

A total of 294 subjects with mild COVID-19 were randomized in a 1:1 ratio to receive either azelastine (1 mg/mL) nasal spray plus standard supportive care or a matching placebo nasal spray plus supportive care using a computer-generated randomization scheme (Supplementary Table S3).

The randomization was simple and there was no restriction on randomization, such as blocking and block size. All participants, care providers, and assessors were blinded after assignment to interventions. Randomization and blinding were achieved using unique serial numbers and coded treatment kits, along with sealed emergency envelopes containing treatment sequences. An independent biostatistician (randomizer) was involved in the generation of the random allocation sequence. Individual sites were responsible for the enrollment of participants. The investigational products were packed and labeled by the Sponsor and were assigned to the participants.

### 2.4. Procedures

After obtaining the informed consent, a thorough clinical history, physical examination, measurement of vital signs, and laboratory parameters including SpO2 levels were conducted for evaluation and eligibility assessment.

Samples for RATs were collected for the detection of COVID-19. All subjects detected positive on the RAT were enrolled and the treatment was initiated on the same day, as per the randomization schedule. Simultaneously, subjects’ nasopharyngeal swabs (for RT-PCR test as confirmatory diagnosis of COVID-19) were collected on the same day. The RT-PCR-positive subjects, regardless of symptoms, were continued in the study; RT-PCR-negative subjects were considered a screen failure. Subjects were randomly assigned to one of the two treatment groups and given a blinded study medication with instructions on how to self-administer each dose. The nasal spray bottles for both the groups were identical. The azelastine formulation included 1 mg/mL of azelastine hydrochloride (i.e., 0.1%; one puff of the spray contains 0.14 mg azelastine hydrochloride) in a solution containing various excipients (disodium edetate, hypromellose, disodium phosphate dodecahydrate, citric acid sodium chloride, and water), while the visually identical placebo had the same excipients minus the active drug.

The study comprised 6 visits over a period of 30 days. Visit 1 (day 1) serves as the screening/baseline/treatment visit, followed by subsequent visits: Visit 2 (days 3 + 1), Visit 3 (days 6 + 1), Visit 4 (days 11 + 1—End of Treatment [EOT]), and follow-up visits: Visit 5 (days 15 + 1), and Visit 6 (days 30 + 1—End of Study). In the case of persistent symptoms, another follow-up visit was to be scheduled on day 60 (±1 day).

The clinical condition of participants was assessed by the investigators using the WHO 11-category ordinal score ranging from a score of 0 to 10, classifying the subject’s status as uninfected, mild, moderate, or severe. Additionally, the alteration in subject status was displayed as the variance between the temperature and blood SpO2 measurements at each visit compared to their respective baseline measurements.

The treatment phase spanned 11 days, and data for quantitative RT-PCR, severity of symptoms, and safety assessment including adverse events (AEs) and change in laboratory parameters like blood oxygen levels and temperature were collected on day 1, day 3, day 6 and day 11. The study procedures and assessments schedule are provided in (Supplementary Table S4).

### 2.5. Efficacy Outcomes

The study’s primary endpoints focused on assessing the rate of COVID-19-related hospitalization in subjects with SARS-CoV-2 infection between the treatment and control groups up to day 11 and determining the reduction in mean virus load via quantitative RT-PCR using nasopharyngeal swabs from baseline (day 1) to day 3, day 6, and day 11.

Secondary endpoints included the proportion of subjects exhibiting a 10-fold decrease in virus load, tracking changes in symptom severity using the MoH checklist, assessing subjects’ status with an 11-category scale proposed by WHO, and determining the proportion of subjects achieving negative conversion of SARS-CoV-2 via RT-PCR as well as time to cure. All the above endpoints were evaluated from baseline [day 1] to day 3, day 6, and day 11.

Exploratory analysis was conducted to analyze subjects with initial cycle threshold (Ct) values below <20 and <25, and assess them based on their vaccination status, grouping them into categories of not vaccinated and those who received one, two, or three doses of the vaccine.

### 2.6. Safety Outcomes

The safety and tolerability of azelastine nasal spray treatment (secondary endpoints) included the assessment of adverse events (AEs) post-randomization until day 30. Safety parameters included AEs, vital signs, physical examination, and laboratory results. The variations from the initial measurements in vital signs and laboratory parameters including the SpO2 and temperature levels were assessed on baseline (day 1) to day 3, day 6, and day 11.

### 2.7. Statistical Analysis

Primary and secondary efficacy analyses were performed on both safety and per protocol (PP) population. All safety analyses were performed using the safety analysis set. For reporting of demographics and frequency of ongoing medical information, a full analysis set (FAS) was used.

The primary outcome of COVID-19-related hospitalization (and not due to comorbid conditions) was analyzed through logistic regression to ascertain the efficacy of the azelastine 0.1% nasal spray. This rate measured the proportion of subjects confirmed with SARS-CoV-2 and subsequently hospitalized at specific visits compared to the total subjects present at that visit.

The second primary endpoint which compared the reduction in mean virus load (area under the curve [AUC] analysis) between two treatment groups was performed using the Chi-square test for categorical variables, and continuous variables were analyzed using analysis of covariance (ANCOVA) with the relevant covariates (baseline, visit, and treatment).

The second primary endpoint (decrease in mean virus load) was measured as the virus load change from baseline and virus AUC based on qRT-PCR values. The change from baseline was determined by subtracting the virus load at follow-up visits from the baseline value, considering log-transformed virus load measurements. The AUC was calculated using a linear trapezoidal rule, incorporating all time points including unscheduled ones from days 1, 3, 6, and 11. All tests were two-sided, maintaining a 5% significance level. Two-group comparisons were examined with the Kruskal–Wallis test. The review of the study data revealed an unexpected baseline imbalance in the outcome variables virus load and log-transformed virus load between the azelastine 0.1% nasal spray and placebo treatment group. Literature related to baseline imbalance of the outcome variables advocated for adjusting models for baseline values due to their increased power in detecting significant treatment effects [17]. The analysis of change-from-baseline method proved ineffective due to the azelastine group’s higher virus load at baseline. A longitudinal ANCOVA provided treatment effect estimation, but computing within-group effects while including the baseline as a covariate required preprocessing by centering both baseline and follow-up measures with the baseline median. This approach allowed for interpreting adjusted means (LSMeans) directly as mean differences from the baseline, supporting within-group effect computation [18]. Thus, adjusted baseline values were centered at zero for both groups to assess within- and between-treatment significance. The longitudinal ANCOVA model’s fixed effects were used to simulate normal random samples for treatment effects and variances, transforming outcome variables as needed.

Safety analyses were carried out on the entire set of randomized participants who had been administered a minimum of one dose of the study drug or placebo (Safety Analysis Set) and included AEs, vital signs, physical examinations, and laboratory findings. Descriptive statistics were used to summarize vital signs and laboratory data for continuous parameters.

All analyses were performed using the statistical software package SAS^®^ version 9.4 or higher (SAS Institute Inc., 100 SAS Campus Drive, Cary, NC 27513-2414, USA). No interim analysis was planned.

The sample size was calculated based on the viral load mean values observed in a previously performed phase II study (CARVIN). The viral load mean values observed in the CARVIN study for azelastine 0.1% group at baseline and day 11 were 6.52 log_10_ copies/mL [±1.94 Standard deviation (SD)] and 2.36 log_10_ copies/mL (±2.12 SD), respectively. Corresponding values noted in the control group were 6.30 log_10_ copies/mL (±2.11 SD) and 2.75 log_10_ copies/mL (±1.82 SD), respectively. A two-sided α = 0.05 and a power of 80%, with a sample size of 126 subjects per treatment group was calculated. Anticipating a drop-out rate of 15%, the aim was to randomize 290 subjects in total (145 subjects per treatment group) to result in 126 subjects per group completing the study and being eligible for analysis.

## 3. Results

### 3.1. Subject Disposition

The study was conducted at 10 sites in India commencing on 27 September 2022 (first participant’s first visit) and concluding on 16 May 2023 (last participant’s last visit). A total of 294 subjects were screened. All screened subjects were randomized in a 1:1 ratio to azelastine 0.1% nasal spray or placebo group. Of the 294 subjects, 251 (85.4%) completed the study and were included in the safety population. A total of 43 (14.6%) subjects discontinued from the study and did not receive even one dose of the study medication. The reasons for discontinuation were “a negative RT-PCR screening result reported post-randomization” in 22 (15.2%) subjects in the azelastine 0.1% nasal spray group and 19 (12.8%) subjects in the placebo group, followed by “withdrawn consent” in one subject (0.7%) in the placebo group and “subject considered as non-evaluable” in 1 subject (0.7%) in the azelastine 0.1% nasal spray group.

**Figure 1:**
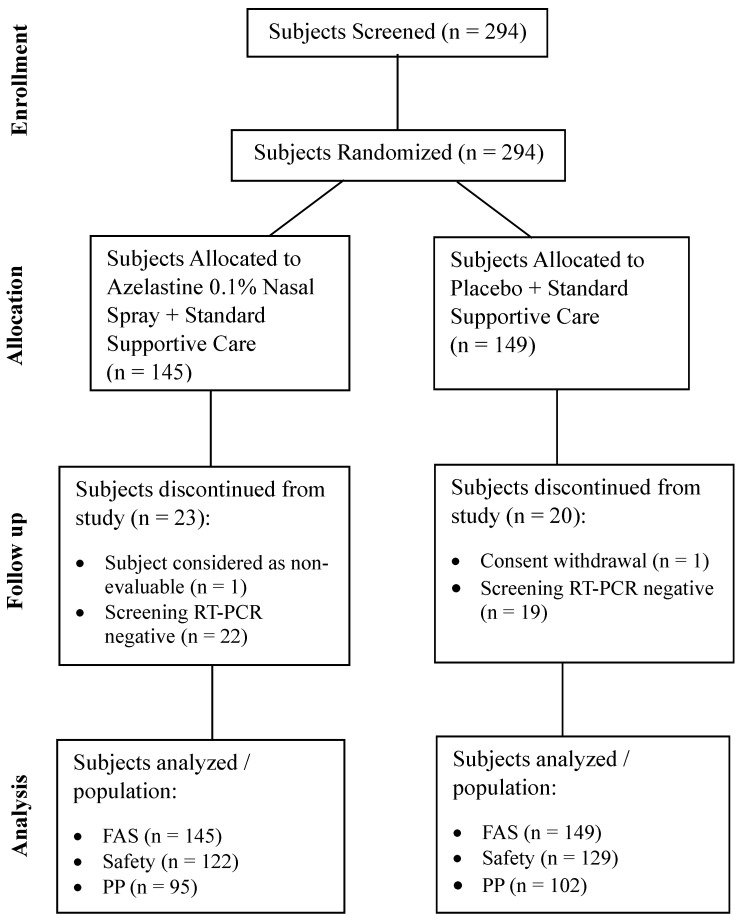
Study Trial Disposition. Abbreviation: FAS = Full Analysis Set; PP = Per protocol; RT-PCR = Reverse Transcriptase Polymerase Chain Reaction; FAS: FAS Population included data from all randomized subjects regardless of the treatment actually received; Safety: All subjects who received at least one dose of randomized IMP, or Placebo and for whom any post-dose data were available included in the safety analysis set. Per Protocol: All subjects from safety analysis set completing the study without major protocol deviations and had their baseline RT-PCR report as positive were included in the PP analysis set.

Of 251 subjects of the safety population, 54 (21.5%) subjects reported at least one major protocol deviation/violation, hence a total of 197 (78.5%) subjects were included in the PP population. Of those 197 subjects, 95 (48.2%) subjects belonged to the azelastine 0.1% nasal spray treatment group, and 102 (51.8%) subjects belong to the placebo group (Figure 1). Mean treatment compliance was >90% and comparable between both groups.

### 3.2. Subjects’ Baseline Characteristics

Both groups had comparable baseline and demographic characteristics, thus excluding a systematic bias and allowing a very good comparability of treatment effects. Of the 251 subjects included in the safety population, 153 (61.0%) were male, the mean (SD) age of all subjects was 39.9 (15.11) years, and all the subjects (100.0%) were Asian (Table 1). Two doses of COVID-19 vaccine were received by 38.5% of subjects in the azelastine 0.1% group and 34.1% in the placebo group. The frequency of ongoing medical information (subject health information) in FAS was similar between the two groups [14 subjects (9.7%) in the azelastine 0.1% nasal spray treatment group vs. 13 subjects (8.7%) in the placebo group]. The most frequent medical history of subjects by system organ class (SOC) and preferred terms (PTs) were hypertension (4.8%) and diabetes mellitus (3.4%) (Supplementary Table S5).

### 3.3. Efficacy

#### 3.3.1. Rate of COVID-19-Related Hospitalization

The primary efficacy endpoint was hospitalization due to COVID-19 infection (and not due to comorbid conditions). There was no incidence of COVID-19-related hospitalization in subjects with SARS-CoV-2 infection in both treatment groups.

#### 3.3.2. Viral SARS-CoV-2 RNA Load Reduction

The second primary endpoint was to compare the reduction in the mean virus load between the two treatment groups. On the log-transformed scale, the mean virus load value (genEq/mL log_10_) at baseline was higher in the azelastine 0.1% nasal spray treatment group as compared to that in the placebo group (log_10_ 6.32 vs. log_10_ 6.20). The log-transformed mean virus load was adjusted to median and then corrected at zero. There was a significant reduction in the mean virus load in both groups during the 11 treatment days as compared with baseline. The reduction in virus load in the azelastine 0.1% nasal spray group was significantly higher than the reduction in the placebo group at day 11 (log_10_ 5.93 vs. log_10_ 5.85, *p* = 0.0041). Similar results were observed at day 3 (log_10_ 2.80 vs. log_10_ 2.62, *p* < 0.0001) and day 6 (log_10_ 5.05 vs. log_10_ 4.94, *p* = 0.0013) (Table 2).

Evaluation of the AUC value based on simulated log-transformed virus load values showed that the azelastine 0.1% nasal spray treatment group exhibited a greater AUC value 1014.960 ± 35.2527, referring to a greater decrease in viral load, compared to the placebo group with an AUC value of 982.977 ± 31.6776 (*p* < 0.0001) (Table 3).

The median time to cure was similar between both groups (225 h; hazard ratio and 95% Confidence Interval [CI]: 0.32 [0.83, 2.32]; *p* = 0.2161) (Supplementary Table S6).

### 3.4. Exploratory Analysis for Robustness of the Primary Endpoint

Robustness of the primary efficacy endpoint (decrease in mean virus load) was checked by exploratory analysis. Subgroups (i.e., initial Ct value < 20 and <25 as well as based on vaccination status [not vaccinated and one/two/three dose(s) of vaccine]) were also checked by exploratory analysis. There was a significant reduction in mean virus load in both treatment groups at day 3, day 6, and day 11 when compared to baseline (*p* < 0.0001) for all subgroup populations (initial Ct value < 20 and <25; vaccination status: not vaccinated and one/two/three dose of vaccine). Additionally, exploratory analysis was also performed on the PP population. Results of the efficacy endpoints (decrease in mean virus load and AUC assessment for virus load) with the PP population were comparable to the results obtained in the safety population which indicated that there was no impact of protocol deviations/violations on the efficacy analysis results, i.e., superior efficacy of treatment (azelastine 0.1% nasal spray) over control (placebo nasal spray) is supported through this sensitivity analysis.

#### 3.4.1. Subjects Demonstrating a 10-Fold Decrease in SARS-CoV-2 Virus Load

The proportion of subjects that demonstrated a 10-fold decrease in virus load of SARS-CoV-2 was comparable at all subsequent visits between both groups (Supplementary Table S7).

#### 3.4.2. Subjects with Negative Conversion of SARS-CoV-2 RT-PCR

The proportion of subjects with negative conversion of SARS-CoV-2 RT-PCR was increased gradually at all subsequent visits when compared to baseline in both groups. The proportion of subjects with negative RT-PCR results was comparable between the two groups at day 3, day 6, and day 11. More than 90% of subjects were found negative in RT-PCR test results at day 11 in both treatment groups (Supplementary Table S8).

#### 3.4.3. Change in Symptom Severity (Based on MoH FW Checklist)

The analysis of symptoms severity (symptom score based on MoH FW checklist) in both groups at baseline showed that the patients were predominantly mildly symptomatic (i.e., anorexia, cough, diarrhea or gastrointestinal upset, fever, headache, loss of smell, loss of taste, malaise, nausea, sore throat or throat irritation, vomiting, and weakness). During the treatment, both groups showed clear improvements in the severity of symptoms, and the majority of symptoms were resolved at day 11 in both groups. However, azelastine 0.1% nasal spray treatment was associated with significant improvement in fever (*p* = 0.0046), weakness (*p* = 0.0012), and hypoxia (*p* < 0.0001) compared to the placebo group.

None of the subjects showed symptoms of mild COVID-19, as outlined by the MoH FW, Government of India, during Visit 6 (day 30 ± 1 day). Therefore, no further follow-up visit (to be scheduled on day 60 (±1 day)) had to be arranged.

#### 3.4.4. Change in Subject Status (Based on the WHO’s 11-Category Ordinal Score)

The subject status was assessed using an 11-category ordinal score as proposed by the WHO which allows classification of the subject’s status as uninfected, mild, moderate, or severe (Score: 0 to 10). The mean ordinal score proposed by the WHO’s clinical progression scale was similar at baseline between the two groups (Score 2). The mean ordinal score was decreased significantly (*p* < 0.0001) at day 3, day 6, day 11, and day 15 in both groups as compared with baseline. At day 6, the mean ordinal score was almost “0” (0.4 in both groups) which reflects that there was no viral RNA detected and there was a clear improvement in subject’s clinical status (Supplementary Table S9).

### 3.5. Safety

The number of subjects that reported at least one adverse event (AE) and overall incidence of AEs were similar between the two groups. A total of 79 subjects (31.5%) experienced at least one AE (total incidence of AEs = 99). A total of 39 subjects (32.0%) in the azelastine 0.1% nasal spray treatment group and 40 subjects (31.0%) in the placebo group reported 48 and 51, AEs respectively. All AEs were considered as treatment-emergent adverse events (TEAEs) in both groups. All 39 subjects (32.0%) in the azelastine 0.1% nasal spray treatment group reported only mild AEs. One subject (0.8%) in the placebo group reported a moderate AE in the study and the remaining 40 subjects (31.0%) reported mild AEs. In both groups, all the AEs were not related to the investigational treatment. There was no action taken with study medication due to AEs and all AEs were recovered/resolved in both treatment groups. There was no serious adverse event (SAE) reported in the study (Supplementary Table S10).

The most common AEs reported by the subjects (≥5%) in both groups were asthenia in 28 (11.2%) followed by nausea in 21 (8.4%), myalgia in 18 (7.2%), and headache in 16 (6.4%) subjects (Table 4). There was no death or other significant AE reported in either of the treatment groups.

There were no clinically significant changes in vital signs (i.e., diastolic blood pressure, pulse rate, respiration rate, and systolic blood pressure) at post-baseline visit when compared to baseline in both treatment groups. However, clinically significant changes were observed in SpO2 which increased gradually at post-baseline visits (day 3, day 6, day 11, day 15, and day 30) and temperature decreased at post-baseline visits (day 3, day 6, and day 11) when compared to baseline visit (day 1) in both groups. The mean change in SpO2 at day 11 from baseline was identical in both groups (1.0% in both groups). Similarly, the mean change in temperature at day 11 from baseline was comparable between the two groups (−2.1 °F in the azelastine 0.1% nasal spray treatment group and −2.2 °F in the placebo group).

There were no clinically relevant trends observed in the majority of hematology, biochemistry, and urinalysis laboratory parameters at day 11 and day 15 when compared to baseline in both groups. The majority of the subjects in both groups had either normal or abnormal clinically not-significant laboratory results at day 11 and day 15. Overall, based on the safety results of the study it was concluded that azelastine 0.1% nasal spray was safe and well-tolerated by the subjects.

## 4. Discussion

COVID-19 starts with the infection of the nasal mucosa and the nasopharynx; therefore, application of locally acting antiviral agents appears to be particularly promising. The obtainability of a self-administrable nasal spray may reduce subsequent viral transmission and thus offers great advantage for the community [17]. For instance, nitric oxide (NO) and the preventive application of a hydroxypropyl methyl cellulose (HPMC) nasal spray have shown to reduce the viral load in adult subjects with mild COVID-19 infections [18,19]. Meanwhile, NO and HPMC nasal sprays appear to act via rather unspecific mechanisms—HPMC by forming a physical barrier on the mucosal surface and NO by nitrosylation of proteins—the well-established antihistaminic OTC-drug azelastine appears to exhibit pharmacological antiviral properties via specific interactions with viral and host target proteins. So far, azelastine has been suggested to inhibit the entry of SARS-CoV-2 into the nasal mucosa by binding to the ACE-2 receptor, furthermore by inhibiting the main protease of SARS-CoV-2 as well as the host cell’s sigma-1 receptor, therewith facilitating both viral entry and replication-inhibiting effects [11,12]. Azelastine was furthermore found to inhibit inflammation and intracellular adhesion molecule-1 (ICAM-1) upregulation induced by viral infections, effects that may contribute to the antiviral activity observed also against non-coronaviruses [14].

In the present study, subjects were eligible for treatment after confirmation of a positive RT-PCR test, and if enrolled no later than 48 h after swab sampling. It is anticipated that virus load is at its peak during the initial phase of the disease, considering the current study setting wherein the treatment with azelastine started during the symptomatic phase of the disease; this scenario corresponds to current COVID-19 treatment regimens (e.g., with monoclonal antibodies or antiviral substances) which are usually started at ≤5–7 days upon the start of symptoms, and are still efficacious. Indeed, the strongest decrease in viral load was seen in this trial within the first days of treatment with azelastine nasal spray, as was observed already previously [15]. Reduction of viral load within the nasopharyngeal area is the proposed mode-of-action of azelastine nasal spray, but a clinical benefit of this surrogate parameter, including improvement in symptoms, remains to be demonstrated.

The study reported no incidence of COVID-19-related hospitalization in both treatment groups up to day 11, indicating overall rather mild courses of the disease in this trial. The outcome of the primary efficacy endpoint in this study is far better than the study conducted by Sinha et al. [20], where nine (1.48%) molnupiravir plus standard-of-care-treated and twenty-six (4.26%) just standard-of-care-treated subjects required hospitalization. Similarly, in a phase III, double-blind study conducted by Jayk Bernal et al. [21] in non-hospitalized, unvaccinated subjects with mild-to-moderate, laboratory-confirmed COVID-19 infection, the risk of hospitalization for any cause of death through day 29 was lower with molnupiravir (28/385 [7.3%]) than with placebo (53/377 [14.1%]).

The study population in the current study had an initial mean virus load of log_10_ 6.26 ± 1.16 genEq/mL which was less than more recently reported SARS-CoV-2 virus load values, and the rate of negative testing increased earlier as compared to the first phase II clinical trial investigating the efficacy of azelastine nasal spray [15]. This observation may be explained with a) the evolution of the virus (while the alpha variant was dominating during conduction of the referenced phase II clinical trial [15], omicron variants dominated during this clinical trial); b) very likely with the much higher rate of preceding infections and vaccinations, causing an earlier and specific antiviral immune-response in patients included into this trial; and c) potentially also due to the different populations included into both studies (dominantly European vs. Asian populations, respectively). Self-administered azelastine 0.1% nasal spray in this study had a statistically significant greater mean reduction in SARS-CoV-2 RNA log_10_ copies/mL over the 11 days of treatment compared to placebo. The results of mean reduction in virus load observed in this study are in line with the results of Tandon et al. [22] where self-administered NO nasal spray (six times daily as two puffs per nostril for seven days; 0.45 mL solution/dose) was associated with a significantly better reduction in the mean virus load throughout the treatment period compared to subjects treated with a placebo. Similarly, in the phase II CARVIN study, azelastine nasal spray 0.1% three times daily for 11 days of treatment was associated with greater virus load reduction compared to placebo (*p* = 0.007) [15]. While the NO nasal spray was administered six times daily, an azelastine 0.1% nasal spray was applied three times daily in the study of Klussmann et al. [15] and five times daily in the reported study, based on a pharmakometric analysis of the CARVIN data, indicating a greater benefit of a five-fold application per day [23].

In the present study, the mean AUC assessment for virus load over the 11-day treatment period was significantly greater in the azelastine 0.1% nasal spray treatment group as compared to the placebo group (1014.960 vs. 982.977, *p* < 0.0001). The results (azelastine 0.1% group AUC value: 24.14 ± 13.12 vs. placebo group AUC value: 18.89 ± 4.70, *p* = 0.007) of the earlier phase II CARVIN study [15] were coherent with the present study.

Throughout the treatment, there were noticeable improvements in symptom severity for both groups in this trial, with most symptoms resolving by day 11. At day 11, the mean ordinal score proposed by WHO was 0.1 in both groups which proves that there was no viral RNA detected. This underlines the overall mild course of disease in the observed population and could be cautiously interpreted as an indication of a fundamental decrease in the severity of SARS-CoV-2 infections.

This is further supported, since in the present study, over 90% of subjects in both groups tested negative for RT-PCR by day 11, which is a much higher rate than observed in the previous phase II study [15], wherein the negative PCR results appeared earlier and more frequently in the azelastine treated groups, with 48.2% of patients exhibiting a negative result by day 11 in the 0.1% azelastine group versus only 23.1% of the patients in the placebo group.

Future research approaches of locally applied antiviral agents could therefore focus on the prevention of SARS-CoV-2 infections rather than on reducing the symptoms of the already increasingly mild courses of the disease, considering that even after mild acute infections there is still a high rate of long-lasting COVID symptoms.

Generally, treatment with azelastine appeared safe in SARS-CoV-2-positive subjects: no SAEs or deaths were reported in the current study, and the number of AEs were comparable between groups. These observations are similar to the previous phase II CARVIN study where subjects receiving either azelastine nasal spray or a placebo had comparable AEs with no safety concerns [15]. The comparable compliance between treatment groups (90.8% in azelastine 0.1% nasal spray vs. 92.5% in placebo group) further supported the observation that the tolerability of the investigational medicinal products did not negatively impact treatment adherence.

The present study showed both strengths and limitations. Thus, eligibility criteria were designed carefully to investigate a clearly defined, homogeneous study population of low-risk subjects with a narrow age range. In addition, intervals between swab sampling were short and the overall number of performed RT-PCR tests was high to allow a very close determination of the virus clearance. Unexpectedly, the mean virus load at baseline was higher in the azelastine group as compared to that in the placebo. Baseline viral load adjustment, albeit without alternative and necessary for proper comparison of the groups, might have an impact on the interpretation of our outcome. The participants of this trial were exclusively recruited in India, which limits transferability of the results to the rest of the world’s population. Absence of long-term follow-up data does not allow any conclusion on whether azelastine nasal spray treatment might have an impact on post-COVID-19 conditions, which should be investigated in future research projects. Finally, we cannot rule out the possibility that the placebo contributed to viral clearance, leading to an overall underestimation of azelastine’s treatment effect. Rinsing and diluting effects as well as the formation of a physical barrier (HPMC is one ingredient of the formulation) might have contributed to such effects.

## 5. Conclusions

Overall, no incidence of COVID-19-related hospitalization, accompanied by significant reduction in viral load and improvements in symptom severity in this study support the use of azelastine 0.1% nasal spray in subjects tested positive for SARS-CoV-2 with mild symptoms.

## Figures and Tables

**Table 1:** Characteristics of the subjects at baseline (safety population).

Parameter	Statistics	Azelastine 0.1% Nasal Spray + Standard Supportive Care (*n* = 122)	Placebo + Standard Supportive Care (*n* = 129)	*p*-Value Between Treatment	Total (*n* = 251)
Age (Year)	*n*	122	129	0.5684	251
	Mean (SD)	39.3 (14.24)	40.4 (15.93)		39.9 (15.11)
	Median	37.0	38.0		37.0
	Min, Max	19.0, 87.0	19.0, 88.0		19.0, 88.0
Height (cm)	*n*	122	129		251
	Mean (SD)	161.0 (6.99)	160.0 (7.23)	0.3004	160.5 (7.12)
	Median	159.0	157.0		158.0
	Min, Max	148.0, 177.8	147.3, 179.0		147.3, 179.0
Weight (kg)	*n*	122	129		251
	Mean (SD)	67.2 (8.26)	66.2 (9.54)	0.3632	66.7 (8.94)
	Median	67.6	65.9		67.0
	Min, Max	48.0, 84.6	42.7, 85.0		42.7, 85.0
BMI (kg/m^2^)	*n*	122	129		251
	Mean (SD)	26.0 (3.31)	25.8 (3.30)	0.6911	25.9 (3.30)
	Median	26.0	25.7		25.8
	Min, Max	17.9, 36.4	16.9, 33.7		16.9, 36.4
	Gender				
Male	*n* (%)	81 (66.4)	72 (55.8)	0.4669	153 (61.0)
Female	*n* (%)	41 (33.6)	57 (44.2)	0.1060	98 (39.0)
	Race				
Asian	*n* (%)	122 (100.0)	129 (100.0)	0.6586	251 (100.0)
	Ethnicity				
Not Hispanic or Latino	*n* (%)	122 (100.0)	129 (100.0)	0.6586	251 (100.0)
	Smoking Status				
Ex smoker	*n* (%)	1 (0.8)	5 (3.9)	0.1025	6 (2.4)
Never	*n* (%)	121 (99.2)	124 (96.1)	0.8480	245 (97.6)
Ct value at baseline	Mean (SD)	23.39 (3.875)	23.68 (3.634)		23.54 (3.749)
	Median	23.08	23.50	0.5547	23.42
	Min, Max	14.2, 34.9	14.4, 35.3		14.2, 35.3
	Vaccination Status				
Unvaccinated subjects	*n* (%)	24 (19.7)	30 (23.3)	0.4142	54 (21.5)
Vaccinated subjects	*n* (%)	98 (80.3)	99 (76.7)	0.9432	197 (78.5)
Dose 1		98 (80.3)	99 (76.7)	0.9432	197 (78.5)
Dose 2		47 (38.5)	44 (34.1)	0.7532	91 (36.3)
Dose 3 (Booster dose)		4 (3.3)	4 (3.1)	1.0000	8 (3.2)

BMI: body mass index; Ct: cycle threshold; Max: maximum; Min: minimum; SD: standard deviation. Percentages were based on the total number of subjects in each treatment. For continuous data, *p*-values were calculated using *t*-test; for categorical data, *p*-values were calculated using paired *t*-test.

**Table 2:** SARS-CoV-2 RNA viral load (genEq/mL) log_10_ change from baseline (=0) through day 11 in adult COVID-19-infected subjects (safety population).

Visit	Statistics	Azelastine 0.1% Nasal Spray + Standard Supportive Care (*n* = 122)	Placebo + Standard Supportive Care (*n* = 129)	* *p*-Value Between Treatment	Total (*n* = 251)
Baseline (day 1)	Mean (SD)	0 (0)	0 (0)	-	0 (0)
	Median	0	0		0
	Min, Max	0, 0	0, 0		0, 0
Mean CFB through day 3	Mean (SD)	−2.80 (0.274)	−2.62 (0.261)	<0.0001	−2.71 (0.282)
	Median	−2.81	−2.62		−2.71
	Min, Max	−3.5, −2.1	−3.2, −1.8		−3.5, −1.8
	# *p*-value of difference	<0.0001	<0.0001		<0.0001
Mean CFB through day 6	Mean (SD)	−5.05 (0.293)	−4.94 (0.246)	0.0013	−4.99 (0.275)
	Median	−5.04	−4.95		−4.98
	Min, Max	−6.0, −4.4	−5.6, −4.2		−6.0, −4.2
	# *p*-value of difference	<0.0001	<0.0001		<0.0001
Mean CFB through day 11	Mean (SD)	−5.93 (0.221)	−5.85 (0.205)	0.0041	−5.89 (0.216)
	Median	−5.93	−5.87		−5.90
	Min, Max	−6.4, −5.3	−6.3, −5.3		−6.4, −5.3
	# *p*-value of difference	<0.0001	<0.0001		<0.0001

SD: standard deviation; Min: minimum; Max: maximum; CFB: change from baseline; *n*: total number of subjects in the respective treatment group. # *p*-values were calculated using paired *t*-test, * *p*-values were calculated using independent *t*-test. The change from baseline was calculated as “Post baseline—baseline”. The difference between the groups was calculated for Azelastine 0.1% vs. placebo (Azelastine 0.1%-placebo).

**Table 3:** Virus load AUC log_10_ for SARS-CoV-2 (safety population).

Statistics	Azelastine 0.1% Nasal Spray + Standard Supportive Care (*n* = 122)	Placebo + Standard Supportive Care (*n* = 129)	*p*-Value # Between Treatment	Total (*n* = 251)
Mean (SD)	1014.960 (35.2527)	982.977 (31.6776)	<0.0001	998.523 (37.0381)
Median	1015.56	984.46		998.58
Min, Max	907.29, 1110.64	900.16, 1058.96		900.16, 1110.64

SD: standard deviation; Min: minimum; Max: maximum; AUC: area under the curve; *n*: total number of subjects in the respective treatment group. The simulated log-transformed virus load values were used to calculate the AUC. # *p*-value was calculated using the Kruskal–Wallis Test.

**Table 4:** Adverse events by system organ class and preferred term (safety population).

System Organ Class/ Preferred Term	Statistics	Azelastine 0.1% Nasal Spray + Standard Supportive Care (*n* = 122)	Placebo + Standard Supportive Care (*n*= 129)	*p*-Value Between Treatment	Total (*n* = 251)
Gastrointestinal disorders	*n* (%)	14 (11.5)	13 (10.1)	0.8474	27 (10.8)
Nausea	*n* (%)	13 (10.7)	8 (6.2)	0.2752	21 (8.4)
Vomiting	*n* (%)	1 (0.8)	5 (3.9)	0.1025	6 (2.4)
General disorders and administration site conditions	*n* (%)	16 (13.1)	13 (10.1)	0.5775	29 (11.6)
Asthenia	*n* (%)	15 (12.3)	13 (10.1)	0.7055	28 (11.2)
Malaise	*n* (%)	1 (0.8)	0		1 (0.4)
Metabolism and nutrition disorders	*n* (%)	1 (0.8)	0 (0)		1 (0.4)
Decreased appetite	*n* (%)	1 (0.8)	0 (0)		1 (0.4)
Musculoskeletal and connective tissue disorders	*n* (%)	6 (4.9)	12 (9.3)	0.1573	18 (7.2)
Myalgia	*n* (%)	6 (4.9)	12 (9.3)	0.1573	18 (7.2)
Nervous system disorders	*n* (%)	8 (6.6)	10 (7.8)	0.6374	18 (7.2)
Headache	*n* (%)	6 (4.9)	10 (7.8)	0.3173	16 (6.4)
Ageusia	*n* (%)	2 (1.6)	0 (0)		2 (0.8)
Respiratory, thoracic, and mediastinal disorders	*n* (%)	2 (1.6)	3 (2.3)	0.6547	5 (2.0)
Cough	*n* (%)	2 (1.6)	1 (0.8)	0.5637	3 (1.2)
Oropharyngeal pain	*n* (%)	0 (0)	1 (0.8)		1 (0.4)
Throat pain	*n* (%)	0 (0)	1 (0.8)		1 (0.4)
Skin and subcutaneous tissue disorders	*n* (%)	1 (0.8)	0 (0)		1 (0.4)
Pruritus	*n* (%)	1 (0.8)	0 (0)		1 (0.4)

*n*: total number of subjects in the respective treatment group; n: number of subjects in the respective system organ class. System organ class and preferred term are coded using MedDRA v26.0. Percentages were based on number of subjects in respective treatment groups in safety population. *p*-values were calculated using the Chi-square test.

## Data Availability

The data presented in this study are available on request from the corresponding author due to privacy restrictions. The study protocol is available in the Supplementary Materials.

## Supplementary Materials

The following supporting information can be downloaded at https://www.mdpi.com/article/10.3390/v16121914/s1: Table S1: List of IECs or IRBs; Table S2: Inclusion and Exclusion Criteria; Table S3: Randomization Procedure; Table S4: Study Procedures and Assessments Schedule; Table S5: Ongoing Medical Information (Subject Health Information)—FAS Population; Table S6: Time to Cure (Safety Population) ; Table S7: Proportion of Subjects Demonstrating a 10-fold Decrease in Virus Load of SARS-CoV-2 (Safety Population); Table S8: Shift from Baseline in RT-PCR Results Across Visits (Safety Population); Table S9: Change in Subject Status Using an 11-Category Ordinal Score as Proposed by the World Health Organization (WHO) (Safety Population); Table S10: Overall Summary of Adverse Events (Safety Population).
