# Supplementary material for: Azelastine Nasal Spray in Non-Hospitalized Subjects with Mild COVID-19 Infection: A Randomized Placebo-Controlled, Parallel-Group, Multicentric, Phase II Clinical Trial
Source: Viruses. 2024 Dec 13;16(12):1914. doi: 10.3390/v16121914 (PMC11680327; doi:10.3390/v16121914)
Supplement: Supplementary file 1 [file viruses-16-01914-s001.zip › viruses-3312695-supplementary.pdf]

Supplementary Material

Azelastine Nasal Spray in Non-hospitalised Subjects with Mild COVID-19 Infection: A randomised placebo-controlled, parallel-group, multicentric, Phase II clinical trial

Appendix

Table of Contents

Table S1. List of IECs or IRBs 2

Table S2. Inclusion and Exclusion Criteria 3

Table S3. Randomisation Procedure 4

Table S4. Study Procedures and Assessments Schedule 5

Table S5. Ongoing Medical Information (Subject Health Information) – FAS Population 7

Table S6. Time to Cure (Safety Population) 7

Table S7. Proportion of Subjects Demonstrating a 10-fold Decrease in Virus Load of SARS-CoV-2 (Safety Population) 7

Table S8. Shift from Baseline in RT-PCR Results Across Visits (Safety Population) 8

Table S9. Change in Subject Status Using an 11-Category Ordinal Score as Proposed by the World Health Organisation (WHO) (Safety Population) 8

Table S10. Overall Summary of Adverse Events (Safety Population) 10

Table S1. List of IECs or IRBs

Name of Site	Ethics Committee Details	EC Chairperson
Seven Hills Healthcare Pvt. Ltd	Medical Ethics Committee Seven Hills. Seven hills Healthcare Pvt. Ltd. Mumbai Maharashtra 400059	Dr. Mohan Joshi
Lifepoint Multispeciality Hospital	Lifepoint Research- Ethics Committee No. 145, Sr, 1, Mumbai Pune Bypass Rd, near Sayaji Hotel, Wakad, Pune, Maharashtra 411057	Dr. Abhimanyu Makane
Saideep Healthcare and Research Pvt. Ltd.	Institutional Ethics Committee, Saideep Hospital Viraj Estate , Behind Yashwant Colony, Near DSP chowk , Ahmednagar , Maharashtra 414003	Dr. Razia Shaikh
Excel Hospital	Excel Hospital Institutional Ethics Committee. , 1-5-56/29, Old Alwal Rd, beside Bharat Petroleum, Near IG Statue , Banda Basti, Secunderabad, Telangana 500010	Dr. Muruli Mohan
Belagavi Institute Of Medical Sciences	Institutional Ethics Committee BIMS Belagavi Institute Of Medical Sciences, Belagavi Dr B R Ambedkar Road Belagavi (Belgaum) Karnataka - 590001 India	Dr. Kishore Bhatt
D.Y. Patil Medical College	Institutional Ethics Committee DY Patil Medical College D. Y. Patil Medical College Kolhapur 869 E ward Kasaba Bawada Kolhapur Maharashtra - 416006 India	Dr. J L Nagaonkar
Tagore Hospital and Research Center	Tagore Hospital Ethics Committee Tagore Lane, Mansarovar Sector 7, Shipra Path, Barh Devariya, Mansarovar, Jaipur, Rajasthan 302020	Dr. Anil Kumar Gupta
MLB Medical college	Ethics committee, M.L.B Medical college MLB Medical College & Associated Hospital MLB Medical college Kanpur Road Jhansi Uttar Pradesh - 284128 India	Dr. Pankaj Atri
KKasturi Medicare Pvt Ltd	Shah Lifeline Hospital and Heart Institute Pvt. Ltd. Geeta Nagar, Phase-7, Mira Bhayander Road, Near Fly Over Bridge, Mira Road (East) Thane, Maharashtra- 401107 India	Dr. Vaishal Shah
Indus Diabetes and Obesity Centre	Shah Lifeline Hospital and Heart Institute Pvt. Ltd. Geeta Nagar, Phase-7, Mira Bhayander Road, Near Fly Over Bridge, Mira Road (East) Thane, Maharashtra- 401107 India	Dr. Vaishal Shah

Table S2. Inclusion and Exclusion Criteria

Inclusion Criteria	Exclusion Criteria
Subjects must meet all the following inclusion criteria in order to participate in this study:<br>1. Subjects or LAR who provided informed consent prior to initiation of any study procedures.<br>2. Subjects or LAR who understood and agreed to comply with planned study procedures.<br>3. Male or female subjects ≥18 years of age at enrollment.<br>4. Subjects with positive RAT for SARS-CoV-2.<br>5. Willing to provide nasopharyngeal swabs.<br>6. For females: non-pregnant, non-lactating with adequate contraception until Day 11, or females unable to bear children (i.e., tubal ligation, hysterectomy, or post-menopausal (defined as a minimum of one year since the last menstrual period). The contraception methods included oral, intravaginal, transdermal hormonal medicinal drugs or devices containing estrogen and progesterone; oral, injectable or implantable hormonal medical drugs or devices containing progesterone-only; IUD; IUS; bilateral tubal occlusion; vasectomized partner; and/ or sexual abstinence	Subjects meeting any of the following criteria are not eligible for the study:<br>1. Subjects had any contraindication for the use of Azelastine (including hypersensitivity to the active substance or other ingredients).<br>2. Subjects who required hospitalization (social admission was an exception wherein the subject might had mild symptoms requiring quarantine, but no facility was available for the same and thereby hospitalization).<br>3. Subjects with moderate (SpO2 <93%) or severe COVID-19 (SpO2 <90%) based on MoH FW guidelines, Revised on 23 September 2021.<br>4. Any concurrent antihistamine therapy (systemic as well).<br>5. Any concurrent nasal spray or any nasalia including nasal lavage fluid.<br>6. Inability to administer the nasal spray.<br>7. Subjects with nasal structure abnormalities, including nasal ulceration, nasal mucosal erosion, large nasal polyps, and marked septal deviations that significantly interfere with nasal air flow.<br>8. Subjects with alcohol or drug dependence.<br>9. Subjects received any concurrent anti-COVID therapy (including off-label use).<br>10. Where, in the opinion of the investigator, participation in the study was not in the best interest of the subjects, or any other circumstances that prevent the subjects from participating in the study safely.<br>11. Specific vulnerable subjects: subjects who were detained or committed to institutions by law court or by legal authorities, such as psychiatric wards, prisons, or other state institutions.<br>12. Subjects with a history of psychiatric illness.<br>13. Simultaneous participation in other clinical studies or previous participation within 30 days before inclusion.<br>14. Being in any relationship or dependence with the Sponsor, CRO and/or Investigator.

Table S3. Randomisation Procedure

**Randomisation Procedure**
Randomisation were done at individual site level. For each site, a list containing the unique serial number of the patient and the randomly assigned treatment arm were shared with the designated point of contact at URSAPHARM for preparing coded IMP for each patient. The coded IMP were shipped to the designated CRO. The CRO dispatched IMP to participating sites. An Independent Biostatistician (Randomizer) was involved in these activities.

Table S4. Study Procedures and Assessments Schedule

	**Treatment Phase**				**Follow-ups**	
**Schedule (day)**	**Day 1[a]**	**Day 3+1**	**Day 6+1**	**Day 11+1[b]**	**Day 15±1**	**Day 30±1**
**Study Visits[c]**	**Screening/Baseline (Visit 1)**	**Visit 2**	**Visit 3**	**Visit 4 (EoT)**	**Visit 5**	**Visit 6 (EoS)**
Informed consent	X					
Rapid Antigen test (RAT)[d]	X					
Inclusion/Exclusion criteria	X					
Demographics[e]	X					
Medical/Surgical history	X					
Physical examination	X					
Vital signs	X	X	X	X		
Laboratory assessments (Hematology, biochemistry, urinalysis)	X			X	X	
Concomitant medications	X	X	X	X		
Temperature measurement	X	X	X	X		
Measurement of Oxygen Saturation *via* pulse Oximeter	X	X	X	X		
UPT (only for female subjects)	X					
Randomisation	X					
Quantitative RT-PCR Measurement[d]	X	X	X	X		
Study Drug Administration[f]	X	X	X	X		
Standard of Care Administration	X	X	X	X		
Subjects Status (*via* 11-category Ordinal Score)	X	X	X	X	X	
Symptom Severity (*via* MoH FW Checklist)	X	X	X	X		
Safety Assessment (Occurrence of AEs)	X	X	X	X	X	X
Final Assessment						X

Abbreviations: UPT=Urine Pregnancy Test; EoT=End of Treatment; EoS=End of Study; AEs=Adverse Events; MoH FW= Ministry of Health & Family Welfare; RT-PCR=Reverse Transcriptase-polymerase Chain Reaction; RAT=Rapid Antigen Test

a On Day 1 (day of inclusion of the subject), at least two applications of the nasal spray per nostril were performed.
b On Day 11 (last day of treatment), at least 1 application was performed before the investigator's/designee visit

c The study visits included home/quarantine facility visits done by the site personnel for all study related activities, as the subjects were quarantined. In case the study personnel was not able to visit the home/quarantine facility due to COVID-19 restrictions amid rising coronavirus cases or any other unavoidable reasons, the study related procedures were performed *via* tele-video conferencing.

Note: In case, the study home/quarantine center visits was conducted as a tele-video conferencing, the following data points were not possible to capture and considered as planned protocol deviations: Respiratory rate and blood pressure.

d If the RT-PCR test was positive, (with or without symptoms) the subjects were continued in the study and if found negative, such subjects were considered screen failures and taken off from the study. All cases of clinically significant laboratory abnormalities were documented as part of medical history and followed up for clinically significant worsening and captured as adverse event.

e Date of birth, age, sex, race, and ethnicity were recorded.

f Study drug was administered at screening/baseline visit i.e., Day 1 (if the subject was tested COVID-19 positive *via* RAT) until Day 11 (refer ‘d’ explanation for details). First drug administration was done by the study personnel and the subject was instructed to self-administer the IMP for subsequent treatment days.

Note:

1) Visit 6 was telephonic safety follow-up visit

2) Daily administrations on Days 2-10 was not less than 3 applications per nostril.

Table S5. Ongoing Medical Information (Subject Health Information) – FAS Population

System Organ Class/Preferred Term	Statistics	Azelastine 0.1% nasal spray + standard supportive care (N=145)	Placebo + standard supportive care (N=149)	Total (N=294)
Subjects having at least one ongoing medical information (Subject health information)	n (%)	14 (9.7)	13 (8.7)	27 (9.2)
Cardiovascular system	n (%)	6 (4.1)	9 (6.0)	15 (5.1)
Hypertension	n (%)	6 (4.1)	8 (5.4)	14 (4.8)
Ischemic heart disease	n (%)	0	1 (0.7)	1 (0.3)
Endocrine, Metabolism And Nutrition	n (%)	8 (5.5)	6 (4.0)	14 (4.8)
Diabetes mellitus	n (%)	7 (4.8)	3 (2.0)	10 (3.4)
Hypothyroidism	n (%)	0	3 (2.0)	3 (1.0)
Raised ALT	n (%)	1 (0.7)	0	1 (0.3)

Abbreviations: ALT=Alanine Aminotransferase; FAS=Full Analysis Set
SOC and PT were coded using the latest version of MedDRA v26.0.
Percentages were based on number of subjects in respective treatment in FAS population.

Table S6. Time to Cure (Safety Population)

	Statistics	Azelastine 0.1% Nasal Spray +Standard Supportive Care (N=122)	Placebo + Standard Supportive Care (N=129)
Time to Cure (Hrs)	Median	225.23	225.25
	95% CI	(223.42, 244.53)	(224.67, 244.62)
	25% and 75%-ile	222.88, 247.17	224.20, 248.08
	Range	27.92, 316.95	28.33, 320.43
	Unstratified Analysis p-value (log rank)	0.2161	
	Hazard ratio and 95% CI	0.32 (0.83, 2.32)	

Abbreviations: CI=Confidence Interval
Note 1: Kaplan-Meier time to event analysis with a log-rank test is performed to compare Azelastine 0.1% nasal spray and Placebo.
Note 2: N = Total number of subject in the respective treatment group.

Table S7. Proportion of Subjects Demonstrating a 10-fold Decrease in Virus Load of SARS-CoV-2 (Safety Population)

	Visit	Statistics	Azelastine 0.1% nasal spray + standard supportive care (N=122)	Placebo + standard supportive care (N=129)	Total (N=251)
Proportion of Subjects	Visit 2	n2	120	127	247
		n(%)	69 (57.5)	77 (60.6)	146 (59.1)

	Visit	Statistics	Azelastine 0.1% nasal spray + standard supportive care (N=122)	Placebo + standard supportive care (N=129)	Total (N=251)
Demonstrating a 10-Fold Decrease in Virus Load of SARS-CoV-2	Visit 3	n3	121	128	249
		n(%)	106 (87.6)	116 (90.6)	222 (89.2)
	Visit 4	n4	121	126	247
		n(%)	120 (99.2)	126 (100.0)	246 (99.6)

Abbreviation: SARS-CoV-2=Severe Acute Respiratory Syndrome Coronavirus 2
Note 1: n2, n3, n4 were number of RT-PCR test results positive or negative subjects in the respective treatment group in safety population at Visit 2, Visit 3, and Visit 4 respectively and were used as denominator of % calculation.

Table S8. Shift from Baseline in RT-PCR Results Across Visits (Safety Population)

	Baseline			
	Azelastine 0.1% Nasal Spray + Standard Supportive Care (N=122)		Placebo + Standard Supportive Care (N=129)	
**Visit**	**Negative**	**Positive**	**Negative**	**Positive**
Visit 2				
Negative	0	48 (39.3)	0	47 (36.4)
Positive	0	72 (59.0)	0	80 (62.0)
Visit 3				
Negative	0	93 (76.2)	0	99 (76.7)
Positive	0	28 (23.0)	0	29 (22.5)
Visit 4				
Negative	0	112 (91.8)	0	119 (92.2)
Positive	0	9 (7.4)	0	7 (5.4)

Note 1: Percentages were based on number of subjects in respective treatment in safety population.
Note 2: Baseline was defined as the last value collected before the first dose of study drug

Table S9. Change in Subject Status Using an 11-Category Ordinal Score as Proposed by the World Health Organisation (WHO) (Safety Population)

	Visit	Statistics	Azelastine 0.1% nasal spray + standard supportive care (N = 122)	Placebo + standard supportive care (N = 129)	Total (N = 251)
Subject Status By 11-Category Ordinal Score	Visit 1	N	122	129	251
		Mean (SD)	2.0 (0.09)	2.0 (0.00)	2.0 (0.06)
		Median	2.0	2.0	2.0
		Min, Max	2, 3	2, 2	2, 3
	Visit 2	N	122	129	251

	Visit	Statistics	Azelastine 0.1% nasal spray + standard supportive care (N = 122)	Placebo + standard supportive care (N = 129)	Total (N = 251)
		Mean (SD)	1.2 (0.98)	1.3 (0.96)	1.2 (0.97)
		Median	2.0	2.0	2.0
		Min, Max	0, 2	0, 2	0, 2
	Change from baseline	N	122	129	251
		Mean (SD)	-0.8 (0.99)	-0.7 (0.96)	-0.8 (0.98)
		Median	0.0	0.0	0.0
		Min, Max	-3, 0	-2, 0	-3, 0
		#p-value	<0.0001	<0.0001	<0.0001
	Visit 3	N	122	129	251
		Mean (SD)	0.4 (0.78)	0.4 (0.81)	0.4 (0.79)
		Median	0.0	0.0	0.0
		Min, Max	0, 2	0, 2	0, 2
	Change from baseline	N	122	129	251
		Mean (SD)	-1.6 (0.79)	-1.6 (0.81)	-1.6 (0.80)
		Median	-2.0	-2.0	-2.0
		Min, Max	-3, 0	-2, 0	-3, 0
		#p-value	<0.0001	<0.0001	<0.0001
	Visit 4	N	122	129	251
		Mean (SD)	0.1 (0.32)	0.1 (0.32)	0.1 (0.32)
		Median	0.0	0.0	0.0
		Min, Max	0, 2	0, 2	0, 2
	Change from baseline	N	122	129	251
		Mean (SD)	-1.9 (0.33)	-1.9 (0.32)	-1.9 (0.33)
		Median	-2.0	-2.0	-2.0
		Min, Max	-3, 0	-2, -0	-3, 0
		#p-value	<0.0001	<0.0001	<0.0001
	Visit 5	N	122	129	251
		Mean (SD)	0.1 (0.26)	0.1 (0.28)	0.1 (0.27)
		Median	0.0	0.0	0.0
		Min, Max	0, 1	0, 2	0, 2
	Change from baseline	N	122	129	251
		Mean (SD)	-1.9 (0.28)	-1.9 (0.28)	-1.9 (0.28)

	Visit	Statistics	Azelastine 0.1% nasal spray + standard supportive care (N = 122)	Placebo + standard supportive care (N = 129)	Total (N = 251)
		Median	-2.0	-2.0	-2.0
		Min, Max	-3, -1	-2, 0	-3, 0
		#p-value	<0.0001	<0.0001	<0.0001

Abbreviations: Max=Maximum, Min=Minimum,; SD=Standard Deviation
Note 1: #p-value was calculated using paired t-tests.
Note 2: change from baseline: post baseline-baseline

Table S10. Overall Summary of Adverse Events (Safety Population)

Safety Population (N=251)				
	**Statistics**	**Azelastine 0.1% nasal spray + standard supportive care (N=122)**	**Placebo + standard supportive care (N=129)**	**Total (N=251)**
Overall incidence of AEs	n (%)	48	51	99
**Subjects**				
With at least one AE	n (%)	39 (32.0)	40 (31.0)	79 (31.5)
With Overall Serious AEs	n (%)	0	0	0
With study drug related AEs	n (%)	0	0	0
With study drug related Serious AEs	n (%)	0	0	0
With TEAEs	n (%)	39 (32.0)	40 (31.0)	79 (31.5)
With Serious TEAEs	n (%)	0	0	0
With Study drug related TEAEs	n (%)	0	0	0
**AEs by severity**				
Mild	n (%)	39 (32.0)	40 (31.0)	79 (31.5)
Moderate	n (%)	0	1 (0.8)	1 (0.4)
Severe	n (%)	0	0	0
**TEAEs by severity**				
Mild	n (%)	39 (32.0)	40 (31.0)	79 (31.5)
Moderate	n (%)	0	1 (0.8)	1 (0.4)
Severe	n (%)	0	0	0
**AEs relationship with study treatment**				
Related	n (%)	0	0	0
Not Related	n (%)	39 (32.0)	40 (31.0)	79 (31.5)
**TEAEs relationship with study treatment**				
Related	n (%)	0	0	0

Safety Population (N=251)				
	**Statistics**	**Azelastine 0.1% nasal spray + standard supportive care (N=122)**	**Placebo + standard supportive care (N=129)**	**Total (N=251)**
Not Related	n (%)	39 (32.0)	40 (31.0)	79 (31.5)
**Action taken with the study medication in subjects with TEAEs**				
Not applicable	n (%)	39 (32.0)	40 (31.0)	79 (31.5)
**Outcome of Subjects with TEAEs**				
Recovered/ Resolved	n (%)	39 (32.0)	40 (31.0)	79 (31.5)
Not resolved	n (%)	0	0	0
Resolved with sequelae	n (%)	0	0	0
Fatal	n (%)	0	0	0
Unknown	n (%)	0	0	0
Ongoing	n (%)	0	0	0

Abbreviations: AEs=Adverse Events; TEAEs=Treatment Emergent Adverse Events
Percentages were based on number of subjects in respective treatment groups in safety population
